# Sero-Prevalence of Hemorrhagic Septicaemia in Cattle and Buffalo Population of Indian States Karnataka and Gujarat

**DOI:** 10.3390/vetsci11080386

**Published:** 2024-08-22

**Authors:** Rajeswari Shome, Amit Kanani, Govindraj Gurrappanaidu, Naveen Kumar Gajalavarahalli Subbanna, Nagalingam Mohandoss, Awadesh Prajapati, Kanaka Baskar, Somy Skariah, G. Shanmugam, Snigdha Madhaba Maharana, Kennady Vijayalakshmy, Rahman Habibur

**Affiliations:** 1 The Indian Council for Agricultural Research-National Institute of Veterinary Epidemiology and Disease Informatics, Bengaluru 560064, India; govindaraj.naidu@icar.gov.in (G.G.); gsnaveenkumar93@gmail.com (N.K.G.S.); nagar75@gmail.com (N.M.); awadesh.prajapati@icar.gov.in (A.P.); somyskaria@gmail.com (S.S.); gshanmugam846@gmail.com (G.S.); madhabamaharana192@gmail.com (S.M.M.); 2 Office of Deputy Director of Animal Husbandry, Foot and Mouth Disease Typing Scheme, Polytechnic Campus, Ambawadi, Ahmedabad 380015, India; amit_kanani@hotmail.com; 3 International Livestock Research Institute (ILRI), Block-C, First Floor, NASC Complex, CG Centre, DPS Marg, Pusa, New Delhi 110012, India; v.kennady@cgiar.org (K.V.); r.habibar@cgiar.org (R.H.)

**Keywords:** hemorrhagic septicemia, buffalo, cattle, iELISA, India, risk factors

## Abstract

**Simple Summary:**

Hemorrhagic septicemia (HS) is an acute, fatal, and septicemic bacterial disease of cattle and buffaloes caused by *Pasteurella multocida (P. multocida)*. The present study aimed to carry out HS surveillance through multi-stage random sampling constituting 692 cattle and buffalo serum samples sourced from two Indian states, four districts, eight clusters, 56 epiunits, and 306 households (HHs) by indirect enzyme-linked immunosorbent assay (iELISA). Significantly higher (*p* < 0.0001) iELISA positives were observed in Gujarat compared to Karnataka state. Of the 306 households visited, 9 of 27 epiunits (33.33%) in Karnataka and 24 out of 29 epiunits in Gujarat were iELISA positive (82.75%) and an association was found to be significant (*p* = 0.0002). However, a non-significant association to species, age, or lactation was recorded, although indigenous cattle breeds had a higher HS sero-prevalence compared to crossbreeds. This study highlights important baseline data on HS sero-prevalence in two major milk-producing states of India at different strata.

**Abstract:**

Hemorrhagic septicemia (HS) is a highly contagious and fatal disease of cattle and buffaloes caused by *P. multocida*. Both conventional and molecular methods are applied in parallel for rapid diagnosis of HS outbreaks and the periodical surveillance strategy to identify risk areas for HS is ignored. The current cross-sectional study aimed to estimate sero-prevalence and associated risk factors for HS in cattle and buffaloes in non-vaccinated regions of two Indian states. HS surveillance was carried out through the multi-stage random sampling technique at different strata. The study employed a questionnaire incorporating host factors (species, breed, sex, age, and lactation) and demographic parameters (state, district, block/cluster and village/epiunits, and household). First, two Indian states known for high milk production were selected followed by two districts within each state, subsequently four clusters within each district, finally 5–10 epiunits within clusters and 5–8 households within clusters were randomly selected to collect cattle and buffalo samples. The chi-square/*p* values and maps were prepared to represent disease prevalence and to correlate disease risk factors at different strata. A total of 692 cattle and buffalo serum samples were sourced from two states of the country (Karnataka-285 and Gujarat-407). In the first strata, antibodies to *P. multocida* were high in Gujarat (14.49%, CI: 11.22–18.30) compared to Karnataka (3.85%, CI: 1.94–6.80) with significant (*p* < 0.0001) association between the states. In the second strata, one of the four districts investigated revealed the highest sero-prevalence (18.61%, CI: 13.81–24.24) with statistical significance (*p* = 0.01) between the districts. Among clusters, one out of eight clusters showed the highest sero-prevalence (23.02%, CI: 16.59–30.54) with statistical significance (*p* = 0.03) between the clusters in the third strata. At epiunit level (fourth strata), 9 out of 27 epiunits (33.33%) visited in Karnataka and 24 out of 29 epiunits sampled in Gujarat were sero-positive (82.75%) in iELISA. At the household level, out of 306 HH visited, 40 HH had at least one positive animal (13.07%) and the *p* value between HH in the two states was highly significant (*p* = 0.0002). Chi-square analysis did not find any association of HS sero-prevalence to species, age, and lactation. However, significantly higher (*p* < 0.05) sero-prevalence was recorded in indigenous cattle breeds (16.56%) compared to crossbreeds (6.59%). Various immunoprophylactics and antibiotic therapies are effective against HS, but inappropriate disease reporting and failure to implement adequate vaccination control measures are the gaps identified. The present study highlights the current scenario of HS sero-prevalence in two of the high milk-producing states of India, which will be useful for stakeholders for undertaking the implementation of surveillance and control strategies for the regions.

## 1. Introduction

Hemorrhagic septicemia (HS) is an acute and often fatal disease, which primarily affects water buffaloes, cattle, as well as other domestic and wild mammals, and is caused by Gram-negative bacterium P. multocida [1,2]. The incubation period ranges from 1 to 3 days leading to sudden death without visible clinical signs. In protracted cases, the incubation period can extend up to 5 days or more. Affected untreated animals generally show signs of high fever, respiratory distress, nasal discharge, oedematous swelling of the throat/brisket region, reduced milk yield, and recumbency leading to death [3].

*P. multocida* strains are classified into five capsular types (A, B, D, E, and F) based on the indirect haemagglutination test, 16 somatic serogroups based on the agar gel precipitation test, and eight LPS genotypes (L1–L8) [4]. Serotypes B:2 and E:2 are two common serotypes of P. multocida associated with HS in Asia and Africa, respectively [5,6]. Molecular analysis revealed serotype B:2 with RIRDC ST-122 and multi-host MLST ST-44 association to bovine HS in Southeast Asia [7].

A large number of *P. multocida* B:2 organisms have been isolated from the HS outbreaks from different countries including India [8,9,10,11]. The HS outbreaks typically manifest as catastrophic epizootics in different Asian and African nations leading to huge morbidity and mortality [1]. In India, HS is responsible for the highest mortality among infectious bacterial diseases affecting buffaloes and cattle. It has been estimated to cause economic losses of USD 792 million per year [12]. In another study, based on the high, medium, and low HS incidence scenarios in bovine population, the projected loss for the study state Karnataka was estimated to be 199 million, 149.2 million, and 995.5 million, respectively [13]. As per the recent study, more than 25,000 outbreaks have been recorded in the past three decades in the country [14] and it has emerged as a disease of considerable economic importance where cattle and buffaloes are vital for milk production and draught power. India is classified as Category A in terms of the global impact of HS, meaning that the disease is endemic and of utmost economic importance to the country in India [15].

The risk factors and impact of husbandry practices for the occurrence and spread of HS have not been well defined [16]. The bacterium typically resides in the nasopharynx of cattle, water buffaloes, bison, pigs, and small ruminants as a commensal. Hot and humid weather is a major contributory factor for HS outbreaks [17]. Most cases of HS in cattle and buffaloes occur as either acute or peracute forms and usually diagnosed on the basis of clinical signs and symptoms in field conditions [18]. The infected and recovered animals become carriers because of persistence of organisms in lymphatic tissues of upper respiratory tract and shed the organisms intermittently in nasal discharges [19]. The carrier stage of *P. multocida* in cattle and buffaloes is a very common feature and these carrier animals are a source of infection to other native animals [20,21]. Presence of carrier animals in the farm yards was stated as one of the risk factors for HS and hence the identification of such carrier animals is necessary before introducing them into the farms [14].

Pronounced clinical signs and high case fatality rates are characteristic to HS infected animals. Isolation and molecular assays are performed to detect *P. multocida* in clinical samples [10,22,23,24]. In the recent study, out of 26,305 HS outbreaks reported in India across the study period 1987–2016, Karnataka and Gujarat states have reported a reasonable proportion (~10%) of annual HS outbreaks [14]. Though HS outbreaks are occurring in many regions of the country, fragmented control approaches and under reporting are the gaps identified [25,26]. HS is the second most important bovine disease after FMD and concerted efforts are needed to control and prevent the disease. The current study aimed to generate baseline data to determine HS sero-prevalence and associated risk factors in bovines at two diverse geographically located Indian states, which are among the top 10 milk-producing states in the country.

## 2. Material and Methods

### 2.1. Ethical Approval

The research was granted approval by the Institutional Ethics Committee, ICAR-NIVEDI, Bengaluru (ILRI project, ANSCNIVEDICOP201900200101) and the collection of samples commenced subsequent to obtaining written consent from all the participants.

### 2.2. General Description of the Study Sites

Karnataka state is situated in the Deccan Plateau of India bordered by six states and the Arabian sea to the West and more than 75% of the entire geographical area has arid or semi-arid climate with average annual rainfall of 1248 mm. Gujarat is a state along the western coast of India bounded by Pakistan to the northwest and by the three Indian states of Rajasthan to the north, Madhya Pradesh to the east and Maharashtra to the southeast. The weather in Gujarat is tropical, with hot summers and mild winters and receives an average rainfall of around 900 mm. Dairy farming is an important source of subsidiary income to small/marginal farmers and agricultural laborers in India. This sector supports the livelihoods of over 200 million rural poor farmers according to Ahuja, et al., 2008 [27]. The milk Karnataka Milk Federation (KMF) and Gujarat Co-operative Milk Marketing Federation Limited (GCMMF) collect milk from village level societies and market the milk and milk products through a structured market system. Both states are successful models for co-operative milk procurement and distribution in the country. The dairy development initiatives/schemes are very well implemented to provide continuous and regular employment to marginally poor farmers which resulted in a quantum jump in milk production and these two states are among the top ten milk-producing states in the country. During this enormous improvement and dairy intensification, many diseases such as FMD, HS, mastitis, infertility, and brucellosis created panic among the farmers and veterinary healthcare personnel [13]. Hence, these states were selected to map HS sero-prevalence primarily to aid stakeholders in implementing control measures.

#### 2.2.1. Sample Size Estimation

**Figure 1:**
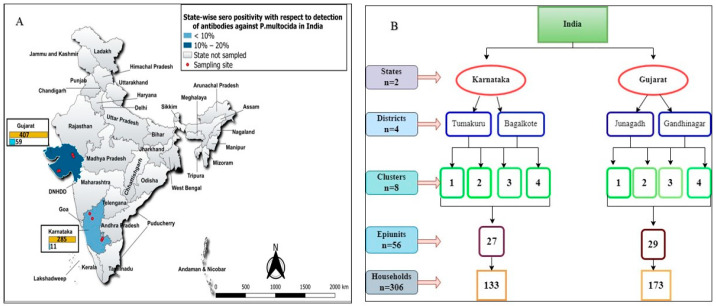
(**A**) India map representing two Indian states in the study; (**B)** Flowchart depicting multi- stage sampling plan.

This study evaluated the sero-positivity and examined the risk factors associated with HS infection in dairy animals during 2022–2023. A multi-stage random sampling method was followed. In the first strata, high milk-producing Karnataka and Gujarat states were selected (Figure 1A). In the second strata, two districts within each state were selected randomly based on cattle and buffalo population upon stakeholder consultation (state Animal Husbandry and Veterinary Department Officials). In the third strata, two blocks/taluks were randomly selected based on availability of primary laboratory/hospital support. In the fourth strata, two veterinary dispensaries/hospitals were selected as clusters and in total eight clusters were selected (four clusters/district). In the fifth strata, 5–10 villages/epiunits and within epiunits cattle and buffaloes were surveyed in 5–8 households (Figure 1B). A local non-governmental organization (NGO) and some leading farmers, including the village headmen, helped in preparing the list of households with a large ruminant population and informed the selected households in advance about the survey. The distribution of the sampled farmers is proportional to the number of cattle and buffalo/households in the identified epiunits. The animal-level prevalence was estimated to be 15–25% at 95% level of confidence and 5% precision; the sample size was calculated to be 327 for Karnataka and 535 for Gujarat (https://epitools.ausvet.com.au/chisq; accessed on 3 February 2024).

#### 2.2.2. Data Collection

Overall, farmers were interviewed on animal characteristics such as animal types (cattle and buffaloes), cattle breeds (crossbred and indigenous), buffalo breeds (Jafarabadi, Mehsana), age (categorized such as 1–2.0, 2.1–4.0, 4.1–6.0, 6.1–8.0, and >8.1 years), and number of lactations (<1, 2, 3, 4, and >5) in 306 households.

#### 2.2.3. Collection of Serum Samples

The project staff and veterinary officers were instructed to collect approximately 5–7 mL of blood aseptically from jugular vein using vaccutainers without anticoagulant (Becton Dickson, Oxford, UK). Separated serum samples from blood clots were transported to ICAR-NIVEDI, Bengaluru, India on ice at a temperature of 2–8 °C. Serum samples received in the institute were centrifuged at 500 g for 3–5 min and separated clear sera was stored at −20 °C until tested. A total of 692 dairy animals from four districts, within which eight clusters consisted of 56 epiunits owned by 306 households from two states, were included in the scope of this study.

#### 2.2.4. Laboratory Procedures

Serological analysis was conducted using the indirect enzyme-linked immunosorbent assay (iELISA) method (Bioassay Technology Laboratory, Jiaxing, Zhejiang Province, China). The IELISA protocol was executed in accordance with the guidelines provided by the manufacturer, adhering to the prescribed test protocol and calculation methods. The values were calculated by comparing the sample well with controls, where the average OD of positive ≥1.00 and the average OD of negative ≤0.10. A cutoff value of the average negative value plus 0.15 was tabulated as per the manufacturer’s recommendation. A sample with an OD value less and greater than the cutoff value were deemed negative and positive, respectively. Sensitivity and specificity of the test were not defined in the kit to estimate apparent and true prevalences in the assayed samples.

#### 2.2.5. Statistical Analysis

Information from the questionnaire was digitized into a Microsoft Excel spreadsheet (Microsoft Corporation) and serological results were interpreted as sero-negative = 0 or sero-positive = 1. The positive animals in epiunits, clusters, and districts within the states were identified. To correlate the degree of association between potential risk factors, the chi-square/*p* value was calculated using Ausvet epitool with a 95% confidence interval (https://epitools.ausvet.com.au/chisq; Accessed on 3 February 2024). The maps were prepared using QGIS software version 3.43.

## 3. Results

### 3.1. Sero-Prevalence of Hemorrhagic Septicemia (HS) at Different Strata

**Figure 2:**
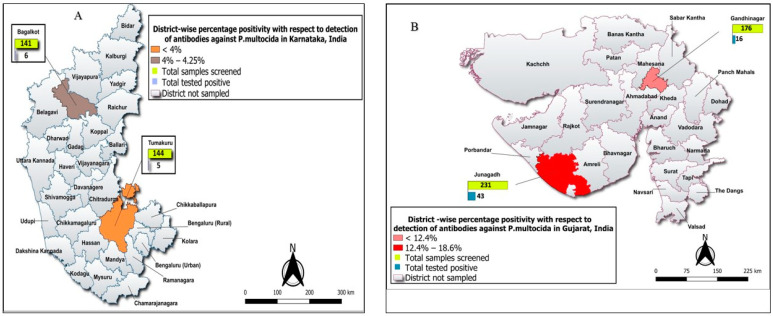
(**A**,**B**) HS sero-prevalence across two districts within Karnataka and Gujarat, India.

A total of 692 cattle and buffalo serum samples were sourced from two Indian states located distantly viz., southern Karnataka and western Gujarat. Out of the 285 samples collected from Karnataka, 259 samples were from cattle and the remaining 26 samples from buffaloes. Similarly, out of the 407 samples collected from Gujarat, 232 samples were from cattle and the remaining 175 from buffaloes. Sero-prevalence of antibodies to *P. multocida* was high in Gujarat (14.49%, CI: 11.22–18.30) compared to Karnataka state (3.85%, CI: 1.94–6.80) and a significant (*p* < 0.0001) association to HS was observed between these top two milk-producing states (Table 1). An investigation into HS sero-prevalence across four districts revealed the highest sero-prevalence in Junagadh district of Gujarat state (18.61%, CI: 13.81–24.24) and between the districts statistical significance (*p* = 0.01) was evident. However, this was not true with the districts visited in Karnataka (Figure 2A,B). Similarly, high sero-positivity was noted in Junagadh cluster among the four clusters of Gujarat state (23.02%, CI: 16.59–30.54) at statistical significance (*p* = 0.03).

**Figure 3:**
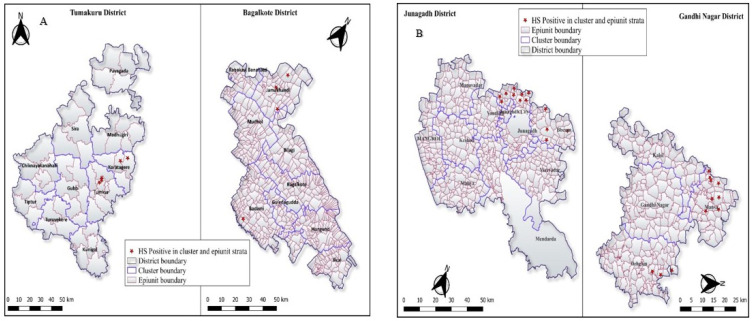
(**A**,**B**) HS sero-prevalence at epiunit level across Karnataka and Gujarat states.

Overall, nine of 27 epiunits (33.33%) visited in Karnataka were positive in the ELISA. Whereas, the majority of epiunits were found positive in Gujarat state (82.75%) (Figure 3A,B). Out of the 306 HH visited, 40 HH had at least one positive animal (13.07%) and *p* value between HH was highly significant (*p* = 0.0002) in two states. In Junagadh cluster, one HH had all four positive animals. Similarly, three animals were positive in four HH and overall, two animals/ HH were found sero-positive across both states (Table 2).

### 3.2. Assessment of Risk Factor for Hemorrhagic Septicemia (HS) Sero-Prevalence

In total, 70 animals were positive out of 692 animals tested (10.12%). Out of 692 samples, cattle constituted the majority with 491 samples accounting for 70.95%, while the remaining 201 samples were from buffaloes (29.04%). Among the 491 cattle samples, 334 (68.02%) were crossbred and 157 (31.98%) were indigenous breeds. Similarly, among the 201 buffalo samples, 162 (80.60%) were crossbred (Mehsana) and 39 (19.40%) were of the indigenous breed Jafarabadi. A total of five variables were examined to evaluate the potential risk factors. Among species, higher sero-prevalence was recorded in buffaloes (10.95%) compared to cattle (9.78%) and a non-significant association to species was recorded. Of the two cattle breeds listed, higher sero-prevalence was recorded in the indigenous cattle (16.56%) compared to crossbred cattle (6.59%) and an association between breeds to HS was significant (*p* = 0.0009). Similarly, among two buffalo breeds sampled, the highest was in indigenous breed Jafarabadi (20.51%) compared to Mehsana (8.64%) at a non-significant level (Table 3).

Under the age category, the highest number of samples [233 (33.67%)] belonged to the 2–4 years age group followed by 221 samples (31.94%) in the 4–6 years age group and 32 samples (4.62%) were from the 1–2 years age group. Similarly, lactation wise, the highest proportion of samples was from the first lactating animals, accounting for 31.12%. Conversely, the lowest number of samples was from animals with over five lactations, comprising 71 samples (10.26%). In the age category, the highest sero-prevalence was in the age group of 1–2 years (18.75%) and similarly, in the first lactating animals (12.04%). Out of the 306 households (HH), 56 HH had vaccinated against FMD and only one farmer had vaccinated against HS. In the HS vaccinated farm, all tested animals were sero-negative.

## 4. Discussion

HS has emerged as the second most frequently reported bacterial disease during the periods of 1990–2000 and 2000–2010 after FMD, causing the highest number of fatalities among large ruminants [13]. In this study, sero-prevalence of HS in cattle and buffalo was conducted at 306 HH comprising 692 animals from two districts each in Karnataka and Gujarat states to evaluate HS burden in these high milk-producing regions of the country.

The disease has a brief incubation period of 12–14 h, approximately 30 h and 46–80 h for subcutaneous infection, oral infection, and natural exposure, respectively [28]. Clinical courses of per-acute and acute cases were 4–12 h and 2–3 days, respectively [29]. Initial symptoms include high fever, loss of appetite, followed by increased respiration rate, nasal discharge, salivation, submandibular oedema, and finally recumbency [30]. In such situations, serological diagnostic assays are of little value. However, the antibody based iELISA test system is used as a screening test for the detection of *P. multocida* antibodies in serum of infected/carrier/recovered animals. Using ELISA, HS sero-prevalence in dairy cows was reported from India and Thailand [25,31]. The sensitivity and specificity values obtained from the ELISA using a coating antigen from *P. multocida* B:2 via heat extraction were higher than that of IHA test [32]. Hence, in the present study, we employed ELISA assay to record HS prevalence. Based on serology, the overall sero-prevalence of HS was found to be 10.12% in two states visited, which is much higher than the reported study from organized farms in other parts of the country [25].

Sero-prevalence of antibodies to *P. multocida* was high in Gujarat (14.49%), compared to Karnataka state (3.85%) and a significant (*p* < 0.0001) association to HS was observed between these states. Investigation into the HS prevalence across districts revealed significantly higher (*p* = 0.01) sero-prevalence in the top milk-producing district (Junagadh) of Gujarat state and in all four clusters visited in the Gujarat state. This clearly indicated wide spread prevalence of the disease in the state. This might be attributed to the lack of vaccination efforts to protect animals against HS and there is a need to revisit both active and passive HS surveillance at cluster, epiunit, and household level in an area. It was interesting to note that in a household, all four animals were sero-positive for HS and some households had three and or two HS positive animals. This represents transmission of the disease within the households which may eventually lead to outbreaks in the absence of vaccination.

Higher HS sero-prevalence was recorded in indigenous cattle than crossbred cattle in Gujarat state and this may be due to the high number of indigenous cattle sampled in the study (*p* = 0009). Also, higher sero-prevalence is attributed to semi-intensive grazing of indigenous cattle unlike the relatively controlled movement in intensive system of rearing practiced for the crossbred animals. Similarly, significantly highest (*p* = 0.065) sero-prevalence was recorded in indigenous buffalo breed Jafarabadi than the crossbred Mehsana (crossbred between Murrah and Surti buffalo breeds). In general, increased susceptibility to HS outbreaks was observed in crossbred animals as they are more susceptible to heat stress during hot and humid climates [32,33]. In the present study, sero-prevalence in buffaloes was found to be non-significantly higher than cattle (*p* = 0.75) but in general, buffaloes are more susceptible to HS [26,32].

Similarly, a study reported high risk of HS morbidity and mortality in small farms and these small holding farms are common at epiunit level (usually maintaining 3–5 animals) as noted in the present study and reported elsewhere [13]. Hence, HS vaccination is equally important for both cattle and buffaloes.

Sero-prevalence was found to be higher in young compared to other age group animals. There is a continuous decrease in sero-prevalence as the age group of animals increased from 2–4 to 2–8 years at non-significant level. A study reported susceptible age group for HS is between 6 months and 2 years [1]. Similarly, sero-prevalence was recorded to be non-significantly higher in first lactating animals compared to other lactations. Both lactation and age of the animals were very well correlated with each other in the current study.

*P. multocida* produce a short duration of immunity in animals up to 4–6 months and the presence of HS antibodies indicates recent infection. As per the farmers and veterinarians feedback, there were no HS-related deaths since the last two months in the regions surveyed. Higher sero-prevalence report in the current study indicates that cattle and buffaloes may be harboring *P. multocida*. These carrier animals may act as a potent source of infection to naïve animals during environmental or transportation stress [28]. Some limitations of the study are that we could not source samples as per the sampling target owing to the lumpy skin disease outbreak during 2021–2023 in India, inaccessibility of the region/s, and the limited logistic support to reach regions was yet another limitation. Isolation of *P. multocida* is a gold standard method for diagnosis of HS, but isolation is tedious and time consuming for a large number samples; hence, antibody detection by ELISA is preferred for surveillance of HS.

The HS disease course is very short and the case fatality rate typically approaches 100% unless the animal is treated very early. Young animals are mainly affected in endemic regions and outbreaks are particularly common during the rainy season in unvaccinated animals. In such situations, locating the endemic areas for vaccination is helpful for preventing the outbreaks [16,34,35,36]. Every year, HS outbreaks have been reported in Gujarat and Karnataka states and hence compulsory vaccination against HS due to *P. multocida* B:2 is a first and foremost step to prevent the annual outbreaks in bovines. The present study on HS sero-prevalence correlates well with the 10% reported occurrence of HS outbreaks among overall reported outbreaks in the country.

## 5. Conclusions

The current study documents a very high sero-prevalence rate of HS disease in Gujarat state compared to Karnataka in cattle and buffaloes. Similarly, within states at different strata, a high sero-prevalence rate for the disease was recorded in few clusters, epiunits, and households. Disease in young animals, indigenous breeds, and buffaloes highlights disease risk for compliance of control measures. The findings underscore regional variations in disease burden, necessitating tailored disease management strategies such as vaccination of the young and heifers and strict surveillance measures for the control of HS in farms in the surveyed regions.

## Figures and Tables

**Table 1:** Detection of antibodies to *P. multocida* at different strata in Indian states.

States Name	Total Positives/Samples, CI 95%	*p* Value between States	Districts Name	Total Positives/Samples, CI 95%	*p* Value between Districts	Clusters Name	Total Positives/Samples, CI 95%	*p* Value between Clusters	Epiunit Positivity, CI 95%	HH Positivity, CI 95%
Karnataka	11/285 (3.85%), [1.94–6.80]	<0.0001 *	Tumakuru	5/144 (3.47%), [1.44–7.92]	0.97	Tumkur	3/83 (3.61%), [0.75–10.20]	0.72	3/9 (33.33%), [7.49–70.07]	3/42 (7.14%), [1.50–19.49]
						Koratagere	2/61 (3.28%), [0.40–11.35]		2/7 (28.57%), [3.76–70.96]	2/35 (5.71%), [0.70–19.16]
			Bagalkote	6/141 (4.25%), [1.58–9.03]		Jamakhandi	4/68 (5.88%), [1.63–14.38]	0.61	3/6 (50.11%), [81–88.19]	4/22 (18.18), [5.19–40.28]
						Badami	2/73 (2.73%), [0.33–9.55]		1/5 (20%), [0.5–71.64]	1/34 (2.94%), [0.07–15.33]
Gujarat	59/407 (14.49%), [11.22–18.30]		Junagadh	43/231 (18.61%), [13.81–24.24]	0.01 *	Junagadh	35/152 (23.02%), [16.59–30.54]	0.03 *	9/9 (100%), [66.37–100]	20/58 (34.48%), [22.49–48.12]
						Bhesan	8/79 (10.12%), [4.47–18.98]		5/6 (83.33%), [35.88–99.58]	6/28 (21.43%), [8.30–40.95]
			Gandhinagr	16/176 (9.09%), [5.29–14.34]		Dehgam	3/55 (5.45%), [4.47–18.98]	0.40	3/6 (50%), [11.81–88.19]	3/25 (12%), [2.55–31.22]
						Mansa	13/121 (10.74%), [5.85–17.67]		7/8 (87.50%), [47.35–99.68]	13/62 (20.97%), [11.66–33.18]

* statistical significant.

**Table 2:** Analysis of sero-prevalence of HS at household level.

States	Districts	No. of HH Surveyed	No. of Animals/HH	One Animal Positive/HH	Two Animals Positive/HH	Three Animals Positive/HH	Four Animals Positive/HH	Total Number of HH Positive	*p* Value between HH in Two States
Karnataka	Tumakuru	77	144	5	−	−	−	5 (6.49%)	0.0002 *
	Bagalkote	56	141	4	1	−	−	5 (8.93%)	
	Total HH	133	285	9	1	−	−	10 (7.52%)	
Gujarat	Junagadh	86	231	15	6	4	1	26 (30.23%)	
	Gandhinagar	87	176	16	−	−	−	16 (18.39%)	
	Total HH	173	407	31	6	4	1	42 (24.28%)	
Total		306	692	40	7	4	1	52	

− represents negative; * statistical significant *p* < 0.001.

**Table 3:** Assessment of the risk factors for sero-prevalence of HS.

Parameters	State	Karnataka	Gujarat	Total Samples (Percentage Positivity)
Age (years)	1–2	0/3	6/29	6/32 (18.75)
	2–4	2/132	16/101	18/233 (7.73)
	4–6	6/118	14/103	20/221 (9.05)
	6–8	2/25	16/117	18/142 (12.68)
	>8	1/7	7/57	8/64 (12.50)
	Total	11/285 (3.85%, CI: 1.94–6.80)	59/407 (14.49%, CI: 11.22–18.30)	70/692 (10.12%, CI: 7.97–12.61)
χ^2^ /*p* value		5.76/0.22	1.40/0.84	5.79/0.22
Species	Cattle	11/259	37/232	48/491 (9.78)
	Buffalo	0/26	22/175	22/201 (10.95)
	Total	11/285 (3.86%, CI:1.94–6.80)	59/407 (14.50%, CI: 11.22–18.30)	70/692 (10.12%, CI: 7.97–12.61)
χ^2^ /*p* value		0.29/0.59	0.67/0.41	0.11/0.75
Cattle breeds	Cross bred	10/237	12/97	22/334 (6.59)
	Indigenous	1/22	25/135	26/157 (16.56)
	Total	11/259 (4.25%,CI: 2.14–7.47)	37/232 (15.64%, CI: 11.48–21.31)	48/491 (9.78%, CI: 7.30–12.75)
χ^2^ /*p* value		0.23/0.63	1.17/0.28	10.94/0.0009 *
Buffalo breeds	Jafarabadi	–	8/39	8/39 (20.51)
	Mehsana	0/26	14/136	14/162 (8.64)
	Total	0/26 (CI: 0–13.23)	22/175 (12.57%, CI: 8.05–18.41)	22/201 (10.95%, CI: 6.99–16.10)
χ^2^ /*p* value		–	0.20/0.66	3.41/0.065
Lactation	1	0/69	26/147	26/216 (12.04)
	2	4/85	10/81	14/166 (8.43)
	3	3/73	7/78	10/151 (6.62)
	4	2/26	7/62	9/88 (10.23)
	>5	2/32	9/39	11/71 (15.49)
	Total	11/285 (3.85%, CI: 1.94–6.80)	59/407 (14.49%, CI: 11.22–18.30)	70/692 (10.12%, CI: 7.97–12.61)
χ^2^ /*p* value		4.47/0.35	6.26/0.18	5.68/0.22

– represents no sample; * significant at *p* < 0.001.

## Data Availability

The data that support the findings of this study are available from the corresponding author upon reasonable request.
